# Development of healthy and sustainable food-based dietary guidelines for the Netherlands

**DOI:** 10.1017/S1368980019001435

**Published:** 2019-07-02

**Authors:** Elizabeth Brink, Caroline van Rossum, Astrid Postma-Smeets, Annette Stafleu, Danielle Wolvers, Corné van Dooren, Ido Toxopeus, Elly Buurma-Rethans, Marjolein Geurts, Marga Ocké

**Affiliations:** 1 The Netherlands Nutrition Centre (Voedingscentrum), PO Box 85700, 2508 CK The Hague, The Netherlands; 2 National Institute for Public Health and the Environment (RIVM), Bilthoven, The Netherlands

**Keywords:** Healthy dietary pattern, Environmentally sustainable diet, Dietary guidelines, Diet optimisation model, Wheel of Five

## Abstract

**Objective:**

To derive healthy and sustainable food-based dietary guidelines (FBDG) for different target groups in the Netherlands and describe the process.

**Design:**

Optimised dietary patterns for children, adolescents, adults and the elderly were calculated using an optimisation model. Foods high in saturated and *trans*-fatty acids, salt and sugar, and low in dietary fibre, were excluded. The dietary patterns resembled the current food consumption as closely as possible, while simultaneously meeting recommendations for food groups, nutrients, maximum limits for foods with a high environmental impact, and within 85 % of the energy requirement. Recommended daily amounts of food groups were based on the optimised dietary patterns and expert judgement.

**Setting:**

The Netherlands.

**Participants:**

FBDG were derived for Dutch people with different ages, genders, activity levels and food preferences.

**Results:**

For most target groups the optimisation model provided dietary patterns that complied with all requirements. For some food groups, the optimised amounts varied largely between target groups. For consistent messages to consumers, the optimised dietary patterns were adjusted to uniform recommendations per target group. Recommendations were visualised in the Wheel of Five. The advice is to eat the recommended amounts of foods according to the Wheel of Five and limit consumption of other foods.

**Conclusions:**

Based on an optimisation model, scientific evidence, information on dietary patterns and expert knowledge, we derived FBDG for different target groups. The Wheel of Five is a key food-counselling model that can help Dutch consumers to make their diets healthier and more environmentally sustainable.

Healthy dietary habits are important for maintaining good health and preventing diet-related chronic diseases^(1)^. Strategies to promote a healthy diet include the development of food-based dietary guidelines (FBDG). FBDG provide advice to the general public on foods, food groups and dietary patterns to provide the required nutrients, prevent chronic diseases and promote overall health while considering culture-specific food preferences^(2,3)^. The methods to develop FBDG differ between countries^(4)^ and have changed over time. More recently, awareness has grown that through adaptations of the daily diet, the environmental impact, like greenhouse gas emissions (GHGE), can be substantially reduced^(5)^. The environmental impact of the diet has been taken into account in some FBDG^(6–8)^.

In 1998, the FAO and WHO published the key scientific considerations for the derivation of FBDG^(9)^. In 2010, these were further specified by the European Food Safety Authority, which advised a stepwise approach that starts with the assessment of relationships between diet and health and ends with graphical representations of FBDG. An important component of the European Food Safety Authority’s approach is testing and optimising FBDG^(2)^. Initially, testing and optimising FBDG was done in an iterative process whereby recommended portions of food groups were modified by experts, using trial and error, until the dietary pattern satisfied the selected constraints^(10–12)^. Constraints could be based, among other things, on nutrient recommendations, current food consumption patterns of the relevant population and cultural factors. In recent years, several countries have used computerised diet optimisation models^(13–15)^ to decrease subjective decision making^(16,17)^ and to improve the fulfilment of the nutrient recommendations^(15)^. Diet optimisation is a mathematical approach that determines the optimal diet given a certain objective function and a set of constraints. In the optimal diet, all constraints are simultaneously achieved if the model provides a solution^(18)^. These constraints are usually intake ranges for nutrients, defined by RDA and safe upper intake levels. The objective function for the optimal diet is often minimising adjustments to the average diet of the target population^(13,15)^ but can also be the lowest environmental impact, price or energetic value^(17,19)^.

The Health Council of the Netherlands (HCNL) derived dietary guidelines based on twenty-nine systematic reviews that summarised randomised controlled trials, prospective cohort studies on nutrients, foods and dietary patterns, and the risk of the top ten major chronic diseases in the Netherlands. Dietary guidelines were formulated for foods and food patterns that lead to health gains, for those food groups for which there was convincing or plausible evidence^(20)^. Therefore, following the guidelines may provide one with dietary patterns lacking energy and sufficient essential nutrients. Moreover, some of the guidelines were rather broad and did not specify quantities, such as ‘Eat legumes weekly’ and ‘Replace refined cereal products with wholegrain products’. In a follow-up process, the Netherlands Nutrition Centre and the National Institute for Public Health and the Environment derived FBDG. In this process, simultaneously the Dutch dietary guidelines^(20)^, the Dietary Reference Values (DRV)^(21)^ and current Dutch consumption patterns^(22–25)^ were taken into account in an optimisation model, and combined with expert judgement.

The aim of the present paper is to describe the process and choices made when deriving the FBDG for the Netherlands and its results: practical recommendations for healthy dietary patterns for different target groups, taking into account aspects of environmental impact of specific foods, visualised in the Wheel of Five.

## Methods

**Fig. 1:**
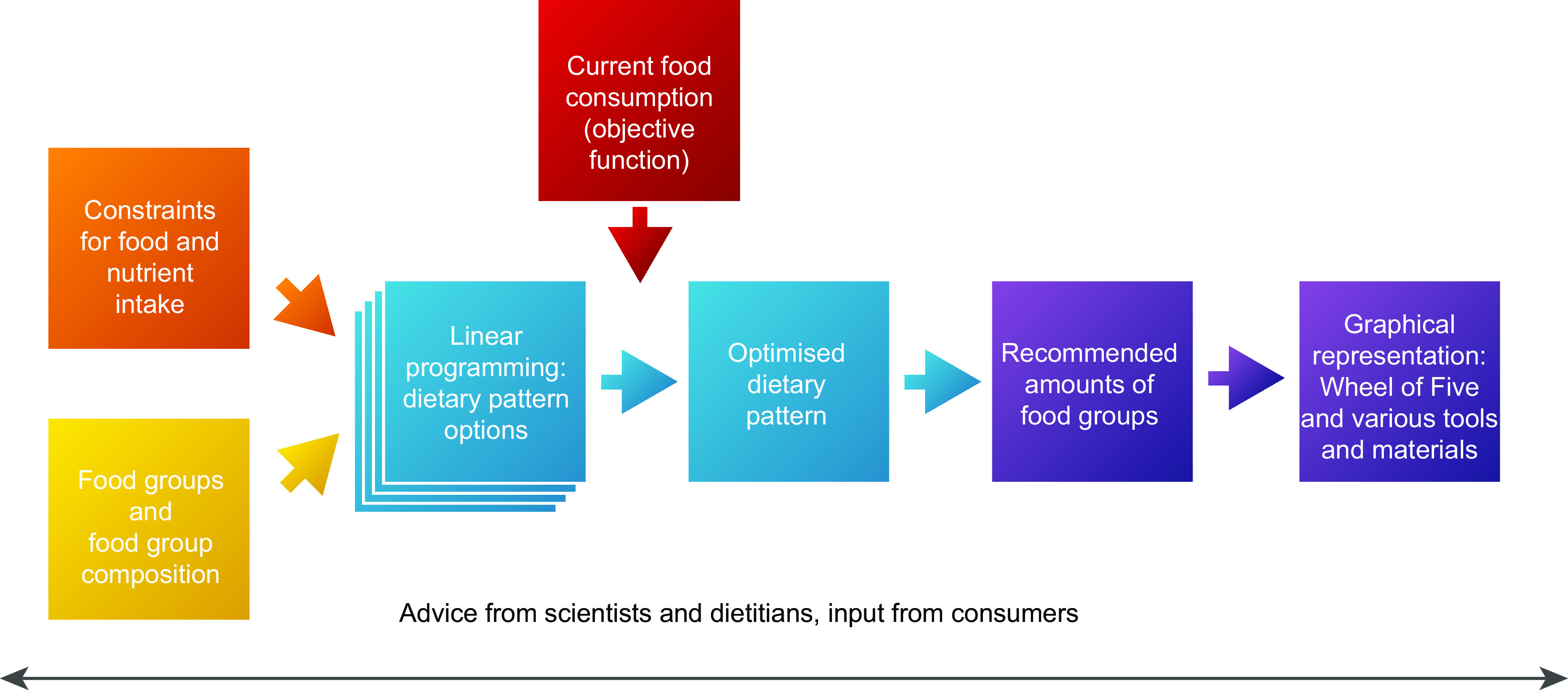
Overview of the development process for food-based dietary guidelines for the general Dutch population (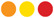, input; 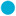, calculations; 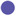, recommendations)

Figure 1 presents a schematic overview of the development process for the FBDG for the Netherlands. We used a dual approach involving calculations and expert judgement. We used a mathematical approach to calculate an optimised dietary pattern for several subgroups among the population, given a set of constraints and objective functions. Constraints were set for food groups based on health effects as described by HCNL^(20)^, considerations with respect to environmental impact in accordance with HCNL guidelines^(20,26)^ and feasibility based on food consumption data^(22–24)^. Minimum and maximum constraints for nutrients and energy were based on the DRV^(21)^ and tolerable upper intake levels^(27)^. The optimised dietary pattern was the pattern closest to the current diet (objective function)^(22–25)^. The results of this approach were translated into FBDG, including a visualisation in the form of the Wheel of Five, web applications and educational materials. External experts representing various disciplines in nutrition science were involved throughout the process, starting with an evaluation of the 2011 FBDG for the Netherlands^(10)^. Additionally, dietitians and consumer groups were consulted for advice and testing concepts.

### Target population groups for the food-based dietary guidelines

The FBDG for the Netherlands were developed for various subgroups among the population that differ in energy and nutrient requirements or dietary habits. First, optimised dietary patterns and subsequently FBDG were derived by gender for the age groups of 1–3, 4–8, 9–13, 14–18, 19–30, 31–50, 51–69 and ≥70 years. Consecutively, FBDG were derived for pregnant and lactating women, for subgroups with a higher activity level, no meat consumption and more non-Western food choices as used by people with Turkish, Moroccan and Surinamese backgrounds living in the Netherlands^(25)^. The FBDG were developed for apparently healthy people with a BMI between 18 and 25 kg/m^2^. For people with underweight, obesity or having a specific disease, individual advice is required by a dietitian who takes account of the individual situation and risks. The FBDG serve as starting point for these advices.

### Food groups for the food-based dietary guidelines

The food groups included in the Wheel of Five were those mentioned in HCNL’s dietary guidelines^(20)^ as being associated with a reduced risk for chronic diseases such as CHD or cancer, like vegetables or wholegrain products, and those that are nutrient-dense and contain essential nutrients^(28)^. Food groups for which HCNL strongly recommends that their consumption be substantially limited or as low as possible were excluded entirely. Examples of such food groups are processed meat and sugary beverages. For other food groups, foods that contain too many nutrients with adverse health effects (SFA, *trans*-fatty acids (TFA), sugar or salt) or too little dietary fibre were excluded from the Wheel of Five. To this end, maximum levels were set for SFA, TFA, sodium and sugar (monosaccharides and disaccharides) and minimum levels for dietary fibre. Per food group, the appropriate levels were established based on the nutrient content^(28)^, the health effect^(20,21)^, the degree of processing, food-group-specific considerations^(29,30)^ and sufficient choice options for consumers^(28)^. The final criteria were reviewed by independent experts not involved in the food industry. As a result of the criteria, parts of some food groups were excluded from the Wheel of Five and the optimisation calculations. For example, refined-grain products were excluded from the cereal group. For foods that were excluded from the Wheel of Five additional recommendations were developed (see below). An overview of the foods and whether or not they were included in the Wheel of Five is shown in Table 1.

**Table 1:** Food group classification and criteria to include or exclude specific foods in or from the Wheel of Five (see Brink *et al*.^(38)^ for detailed information)*,†

Food group	Nutrients for which criteria were set					Additional criteria
	SFA	TFA	Sodium	Sugar (added/total)	Fibre	
Vegetables‡	x	x	x	x		No drinks
Fruit‡	x	x	x	x		No drinks
Potatoes‡	x	x	x	x		
Bread	x	x	x	x	x	
Cereal products (no bread)	x	x	x	x	x	
Legumes‡	x	x	x	x		
Nuts‡	x	x	x	x		
White meat and meat replacements	x	x	x	x		Iron, vitamin B_12_ or thiamin, protein for meat replacements
Red meat	x	x	x	x		
Milk (products) and milk substitutes	x	x	x	x		Calcium, vitamin B_12_, protein for milk substitutes
Cheese and cheese substitutes	x	x	x	x		Calcium, vitamin B_12_, protein for cheese substitutes
Oils, fats and spreads	x	x	x	x		
Non-alcoholic beverages						Only water, tea, coffee without sugar are in the Wheel of Five

TFA, *trans*-fatty acids.

* Food groups fully placed inside the Wheel of Five: fish, eggs. Fish products should consist of at least 70 % fish.

† Food groups fully placed outside the Wheel of Five: processed meat, cold cuts, alcoholic beverages, ready-to-eat meals, sandwiches, soups, sauces, savoury snacks, sweet snacks, savoury bread spreads, sweet bread spreads, miscellaneous.

‡ Subdivision in unprocessed and processed.

### Optimisation calculations

#### Constraints

The basic principles were that recommended amounts of foods in the Wheel of Five deliver 100 % of the essential nutrients and that limited consumption of foods that are excluded from the Wheel of Five is allowed. The first principle could be realised when about 85 % of the total energy was provided by foods in the Wheel of Five. The constraint for energy was therefore set at 85 % of the energy requirement. The energy requirement was the Estimated Average Requirement for individuals with an inactive lifestyle, in order to prevent overconsumption and overweight^(31)^. For adults, the Estimated Average Requirements were calculated based on BMR and a physical activity level^(31)^ of 1·4 for women and 1·5 for men^(32)^. Average weights for BMR estimations were obtained from the Public Health Monitor 2014. The corresponding BMI values were 23·2 kg/m^2^ for men and 22·4 kg/m^2^ for women. The energy requirements for children were based on Dutch growth diagrams^(33,34)^ and literature^(35–37)^.

In addition, minimum and maximum constraints for daily intakes of foods (Table 2) and nutrients (Table 3) were defined. An extensive description of the choices made and the rationale is given by Brink *et al*.^(38)^.

**Table 2:** List of food constraints for adults used in the optimisation calculations in the development of food-based dietary guidelines for the Netherlands

Food group	Minimum	Reason for minimum	Maximum	Reason for maximum
Vegetables (g/d)	200	Health*	–	
Fruit (g/d)	200	Health*	–	
Wholegrain cereals (g/d)	90	Health*	–	
Fish (g/week)	100	Health†	125	Environmental impact║
Legumes (g/week)	65	Health†	135	Feasibility§
Red meat (g/week)	–		Male: 500†,‡ Female: 50th percentile of consumption§	Health†, environmental impact║
Total meat (g/week)	–		50th percentile of consumption§	Environmental impact‖
Eggs (g/week)	–		150	Health†, environmental impact║
Nuts (g/d)	15	Health*	25	Feasibility§
Dairy products (g/d)	300	Health†	75th percentile of consumption§	Environmental impact║

* Quantitative guideline of the Health Council of the Netherlands^(20)^.

† Translation of qualitative guideline of the Health Council of the Netherlands^(20,38)^.

‡ Recommendation of the World Cancer Research Fund^(40)^.

§ Dutch National Food Consumption Survey^(22)^.

║ Based on guidelines of the Health Council of the Netherlands^(20,26)^.

**Table 3:** List of constraints for energy and nutrients used in the optimisation calculations in the development of food-based dietary guidelines for the Netherlands

Nutrient or energy	Minimum	Reason for minimum	Maximum	Reason for maximum
Energy	85 % of the EAR for inactive persons*	Health	85 % of the EAR for inactive persons*	Health
Macronutrients Total fat Total protein Total carbohydrates	Lower value of recommended range†	Health	Higher value of recommended range†	Health
Fatty acids with maximum intake *Trans*-fatty acids SFA	–	–	Recommended maximum intake†	Health
Fatty acids and dietary fibre MUFA α-Linolenic acid Linoleic acid DHA EPA Dietary fibre	Recommended intake†	Health	–	–
Nutrients with AI and UL Calcium Zinc Selenium Copper Iodine Retinol Vitamin B_6_ Vitamin D Vitamin E	AI or RDI†	Health	UL‡	Food safety
Nutrients without UL Phosphorus Iron Potassium Magnesium Thiamin Riboflavin Vitamin B_12_ Vitamin C Folate equivalents Nicotinic acid Retinol activity equivalents Vitamin K	AI or RDI†	Health	–	–
Other nutrients with maximum intake Folic acid	–	–	UL‡	Food safety
Sodium	–	–	Recommended maximum intake§	Health
Alcohol	–	–	Recommended (maximum) intake§	Health

AI, Adequate Intake; UL, safe upper level; EAR, Estimated Average Requirement; RDI, Recommended Dietary Intake; DRV, Dietary Reference Value.

Detailed information on constraints is available in the online supplementary material, Supplemental Table S1.

* Energy intake recommended by Health Council of the Netherlands is the EAR in order to prevent overconsumption and undesirable weight gain^(31)^. Physical activity level value for inactive persons is 1·4 for women and 1·5 for men^(32)^.

† Health Council of the Netherlands’ DRV^(21)^. DRV are given as either RDI or AI, which have the same application.

‡ UL of the European Food Safety Authority^(27)^.

§ Dietary guidelines of the Health Council of the Netherlands^(20)^.

Minimum constraints were set for vegetables, fruit, wholegrain products, unsalted nuts, legumes, dairy products and fish because HCNL showed that consumption of these products is associated with a reduced risk for chronic diseases^(20)^. For vegetables, fruits, wholegrain products and unsalted nuts, HCNL derived quantitative guidelines such as ‘Eat at least 15 g of nuts daily’. These quantities were set as minimum constraints for the specific food groups. For dairy products, fish and legumes, HCNL derived qualitative guidelines. These were translated to minimum constraints taking into account serving sizes and/or current consumption^(22)^, or nutrient provision^(28)^. The HCNL guideline ‘Take a few portions of dairy products daily, including milk or yoghurt’ was translated into a minimum constraint of 300 g, given a serving size of 150 g and the additional HCNL recommendation to maintain the current consumption, which is on average about 300 g/d^(22)^. For fish the minimum constraint was the serving size of 100 g. For legumes the minimum was the amount that delivers the same amount of iron as one serving of meat^(28)^.

**Table 4:** Results of the optimisation calculations for the Dutch food pattern per age and gender. Data are presented as grams per day*

Food group	9–13 years		14–18 years	19–30 years		31–50 years		51–69 years		≥ 70 years	
	M	F	M	M	F	M	F	M	F	M	F
Vegetables	272	389	234	200	561	204	700	246	249	235	271
Fruit	200	200	200	200	200	200	200	200	200	200	200
Bread	214	260	254	275	334	271	245	232	136	195	113
Cereal products	81	0	221	200	9	169	75	125	76	82	132
Potatoes	163	140	193	147	26	137	0	133	93	127	37
Fish and shellfish	18	18	16	15	18	16	18	18	18	18	18
Legumes	19	19	19	19	19	19	19	19	19	19	9
Poultry	43	63	32	31	68	11	68	22	13	30	19
Red meat	46	13	28	28	8	26	8	41	57	70	57
Eggs	21	21	6	19	21	21	21	21	21	21	21
Nuts and seeds	25	25	25	25	25	25	25	22	22	25	15
Milk and dairy products	258	256	285	277	299	267	300	270	252	303	293
Cheese	64	44	47	23	1	33	0	39	48	61	53
Spreadable and cooking fats	36	26	79	96	22	92	22	80	42	54	32
Drinks	487	1194	554	969	3524	1340	3201	1220	1594	1191	1369

M, male; F, female.

* For five age and gender groups (boys and girls aged 1–3 years; boys and girls aged 4–8 years; girls aged 14–18 years), the optimisation model did not deliver solutions that met the Dietary Reference Values for essential nutrients within the goals set for energy.

**Table 5:** Recommended daily amounts of food groups for children in the food-based dietary guidelines for the Netherlands*

Food group	1–3 years	4–8 years	9–13 years		14–18 years		Serving unit and serving size
	M + F	M + F	M	F	M	F	
Daily recommended							
Vegetables	50–100 g	100–150 g	150–200 g	150–200 g	250 g	250 g	50 g
Fruit	150 g	150 g	200 g	200 g	200 g	200 g	100 g
Bread	2–3 slices	2–4 slices	5–6 slices	4–5 slices	6–8 slices	4–5 slices	Slice: 35 g
Cereal products and potatoes†	1–2 servings	2–3 servings	4–5 servings	3–5 servings	6 servings	4–5 servings	Tablespoon of cereals: 50 g Medium potato: 70 g
Nuts and seeds	15 g	15 g	25 g	25 g	25 g	25 g	Handful: 15–25 g
Dairy products	2 servings	2 servings	3 servings	3 servings	4 servings	3 servings	Glass or small bowl: 150 g
Cheese	–	20 g	20 g	20 g	40 g	40 g	For slice of bread: 20 g
Spreadable and cooking fats	30 g	30 g	45 g	40 g	55 g	40 g	Serving of spreadable fat: 6 g Serving of cooking fat: 15 g
Drinks	650 ml	850 ml	1000 ml	900 ml	1300 ml	1000 ml	Glass: 150 ml Beaker: 250 ml
Weekly recommended							
Fish and shellfish	0·5 serving	0·5 serving	1 serving	1 serving	1 serving	1 serving	100 g unprepared fish
Legumes	0·5 serving	1–2 servings	2 servings	2 servings	2–3 servings	2–3 servings	Tablespoon: 60 g
Meat (maximum) of which max red meat	max 250 g	max 250 g	500 g 300 g	500 g 300 g	500 g 300 g	500 g 300 g	100 g unprepared meat
Eggs	1–2 eggs	2–3 eggs	2–3 eggs	2–3 eggs	2–3 eggs	2–3 eggs	Egg: 50 g

M, male; F, female.

* Recommended daily amounts of food groups provide about 85 % of the energy requirement.

† Half of this amount should consist of whole-wheat products each week.

**Table 6:** Recommended daily amounts of food groups for adults in the food-based dietary guidelines for the Netherlands*

Food group	19–50 years		51–69 years		≥ 70 years		Pregnant	Lactating	Serving unit and serving size
	M	F	M	F	M	F			
Daily recommended									
Vegetables	250 g	250 g	250 g	250 g	250 g	250 g	250 g	250 g	50 g
Fruits	200 g	200 g	200 g	200 g	200 g	200 g	200 g	200 g	100 g
Bread	6–8 slices	4–5 slices	6–7 slices	3–4 slices	4–6 slices	3–4 slices	4–7 slices	6–7 slices	Slice: 35 g
Cereal products and potatoes†	4–5 servings	4–5 servings	4 servings	3–4 servings	4 servings	3 servings	4–5 servings	4–5 servings	Tablespoon of cereals: 50 g Medium potato: 70 g
Nuts and seeds	25 g	25 g	25 g	15 g	15 g	15 g	25 g	50 g	Handful: 15–25 g
Milk and dairy products	2–3 servings	2–3 servings	3 servings	3–4 servings	4 servings	4 servings	2–3 servings	2–3 servings	Glass or small bowl: 150 g
Cheese	40 g	40 g	40 g	40 g	40 g	40 g	40 g	40 g	For slice of bread: 20 g
Spreadable and cooking fats	65 g	40 g	65 g	40 g	55 g	35 g	40–50 g	50 g	Serving of spreadable fat: 6 g Serving of cooking fat: 15 g
Drinks	1500 ml	1100 ml	1400 ml	950 ml	1300 ml	900 ml	1500 ml	1500 ml	Glass: 150 ml Beaker: 250 ml
Weekly recommended									
Fish and shellfish	1 serving	1 serving	1 serving	1 serving	1 serving	1 serving	1 serving	1 serving	100 g unprepared fish
Legumes	2–3 servings	2–3 servings	2–3 servings	2–3 servings	2–3 servings	2–3 servings	2–3 servings	2–3 servings	Tablespoon: 60 g
Meat (maximum) of which max red meat	500 g 300 g	500 g 300 g	500 g 300 g	500 g 300 g	500 g 300 g	500 g 300 g	500–675 g 300–385 g	675 g 385 g	100 g unprepared meat
Eggs	2–3 eggs	2–3 eggs	2–3 eggs	2–3 eggs	2–3 eggs	2–3 eggs	2–3 eggs	2–3 eggs	Egg: 50 g

M, male; F, female.

* Recommended daily amounts of food groups provide about 85 % of the energy requirement.

† Half of this amount should consist of whole-wheat products each week.

Additionally, for unsalted nuts, legumes, total meat and red meat, eggs, fish and dairy products, maximum constraints were set. Reasons were a low current consumption (nuts and legumes)^(22)^, a high consumption being associated with increased risk for chronic diseases (eggs and red meat)^(20)^ and a limitation of consumption of animal foods because of environmental aspects (meat, red meat, dairy products, eggs, fish)^(20,26,39)^. For nuts a maximum level was set as the amount that provides the same amount of iron as one serving of meat^(28)^, and for legumes as the amount that is eaten by users on consumption days^(22)^. For eggs, HCNL indicates that a more than average consumption of cholesterol-rich products is not desirable^(20)^. The maximum level for eggs was set at the current consumption^(22)^. For meat and red meat we ambitiously set a maximum level at the 50th percentile of the current consumption^(22)^ in order to limit the consumption of animal products with high GHGE. However, for men the 50th percentile for red meat was over 700 g/week, whereas the World Cancer and Research Fund recommends a maximum of 500 g/week, because high consumption of red meat is associated with increased risk for colon cancer^(40)^. Therefore, this was set as the maximum constraint for men. For dairy products the 50th percentile of the current consumption is meeting the guideline of HCNL, which is associated with a reduced risk for colon cancer^(20,22)^. Additionally, dairy products are important sources of essential nutrients for which the current consumption for some groups lower is than the DRV (like calcium, potassium and vitamin A)^(22,28)^. We therefore chose the 75th percentile of the current consumption as a maximum level. For fish, finally, it was shown that more than one portion of fish per week does not add to reduction of disease risk^(20)^ whereas it does have environmental impact. We chose a maximum taking into account one big serving of 125 g.

The food group constraints for adults are shown in Table 2. For children, the minimum levels for some food groups were extrapolated to lower amounts. The starting point was 75 % of the amount for the 9–13 years age group, 50 % for the 4–8 years age group and 25 % for the 1–3 years age group. This applied to vegetables, fish and legumes. For fruits and wholegrain products, other percentages were used. For nuts and dairy products, the same minimum levels were used for children and adults. More detailed information on the levels and rationale is available^(38)^.

Constraints for nutrients were based on HCNL’s DRV^(20)^ and the European Food Safety Authority’s tolerable upper intake levels^(27)^. The minimum level was set at the level of the recommended intake or adequate intake. The minimum level for vitamin D was set at 3 µg/d, assuming that the rest of the required vitamin D was synthesised in the skin or obtained by dietary supplements^(41)^. The tolerable upper intake level, if applicable, was set as the maximum level. Recommendations for macronutrients were given as a range related to the energy intake^(31)^. For macronutrients, the minimum and maximum levels were based on respectively the lower and upper value of the recommended range. The constraints for nutrients are shown in Table 3 and the online supplementary material, Supplemental Table S1.

#### Nutrient composition of the food groups

The food groups included in the optimisation calculations were those that were part of the Wheel of Five (Table 1). The nutrient and energy contents of each food group were calculated. This was a weighted average of the nutrient values of all currently consumed foods per food group. This information was obtained from the results of the Dutch National Food Consumption Surveys^(22–24)^, which were combined with an extended version of the Dutch Food Composition Database 2013^(28)^. Because the optimised diet should be achievable without choosing fortified products, fortified foods were excluded, except for products for which the Netherlands had made special arrangements with producers. These included products with iodised bakers’ salt and margarines and products used for baking and frying fortified with vitamin A and vitamin D. Additionally, fortified meat substitutes (iron, thiamin, vitamin B_12_) and dairy substitutes (calcium, vitamin B_12_) were included. The weighted mean composition was calculated for five age groups: 1–3, 4–8, 9–18, 19–69 and ≥ 70 years, without sex distinction.

#### Optimisation model

The optimisation calculations were performed with the optimisation model Optimeal^®^ (www.optimeal.info)^(42,43)^, which was modified for this purpose. For each target group, a dietary pattern was generated that complied with the constraints and was as close as possible to the current diet (objective function) for reasons of cultural acceptability. ‘As close as possible’ is defined as minimising the sum of the squared differences (quadratic function) in food group amounts (grams) of the optimised diet and the consumption in the Dutch National Food Consumption Surveys^(22–24)^. A quadratic function was chosen rather than an ordinary linear function to give preference to small changes in more food groups over a large change in one food group; for example, a preference for 20 g difference in three food groups (sum of squared differences = 3 × 20^2^ = 1200), rather than a difference of 60 g in one food group (squared difference = 3600). This approach is in line with our recommendations to improve a dietary pattern by small steps, and not by suddenly introducing major changes.

### Deriving recommended daily amounts for food groups

In order to be able to send consistent messages to consumers, the optimised dietary patterns in grams were converted into recommended daily amounts for food groups in practical quantities and serving sizes for the various target groups. This was done in an iterative process based on expert judgement and took account of the constraints and results of the optimisation calculations and serving size. The result was a recommended dietary pattern for each target group. The nutrient provision was checked for all recommended dietary patterns. If not all DRV for nutrients were met, some adaptions in recommended amounts were made, or specific points of attention for consumer advice were defined.

Feasibility, environmental impact and consistency between the various target groups were also considered. In case that the optimisation calculations yielded no solution, the recommendations were extrapolated from the recommendations for other groups, taking into account their energy and nutrient requirements^(21)^ and current consumption^(22,24)^. Particularly the steps for deriving recommended daily amounts were discussed in expert sessions. The (understanding of the) resulting set of recommendations was tested among dietitians and consumers.

#### Approach for target groups with higher energy requirements and different food preferences

##### Higher energy requirements

For pregnant and breast-feeding women, no recent food consumption data were available. Therefore, the recommendations were based on those for women aged 19–50 years. As pregnant and breast-feeding women have higher nutrient and energy requirements^(21)^, additional recommendations for foods were derived by experts to meet these requirements. These were based on the habitual dietary pattern of this age group^(22)^ and food composition data^(28)^.

The recommended food amounts were set for an inactive population with a physical activity level value of 1·5 and 1·4 for men and women, respectively^(32)^. A physical activity level value of 1·7 was used for more active persons^(32)^, resulting in a higher energy requirement. General recommendations were formulated to meet these extra energy requirements on top of the derived recommended daily amounts for foods.

##### No meat

In order to give practical recommendations to those who prefer to omit meat from their diet, a recommended dietary pattern without meat, but including fish, was derived for all above-mentioned target groups. The basic principle was to replace the recommended amounts of meat with a combination of legumes, nuts and eggs. This was done in a similar way to the iterative process described above.

##### Non-Western dietary patterns

Part of the Dutch population consists of non-Western migrants. The three main groups are people with a Turkish, Moroccan and Surinamese background^(44)^. It was evaluated whether recommendations for the general population were applicable to these groups. Optimisation calculations were performed as described above with some adaptations^(38)^: constraints were adapted to lower energy requirements because of their shorter average height^(25)^ and to recommended amounts for foods for the general Dutch population. Additionally, constraints for some nutrients were excluded for women. Food group composition and current consumption were based on food consumption data for non-Western groups^(25)^. It was evaluated whether the results of the optimisation calculations corresponded with the recommendations for the general population. Specific recommendations were defined for these target groups as required.

### Deriving criteria and recommendations for foods outside the Wheel of Five

As the recommended daily amounts of Wheel of Five food groups cover about 85 % of energy requirements, a limited consumption of foods that are not included in the Wheel of Five is possible. This group includes foods that contain relatively high amounts of salt, sugar, TFA or SFA, or are low in fibre. As foods differ in terms of use (like serving size or eating moment) and composition, we decided to develop generic serving-based recommendations, allowing consumers to supplement their diets according to their own preference. A criteria-based distinction was made between foods low in energy and/or unfavourable nutrients, of which at least three servings could be chosen daily, and foods high in energy and/or unfavourable nutrients that should be chosen by exception. Based upon the difference between the recommended maximum intake of energy and the unfavourable nutrients and their provision within the Wheel of Five food groups, it was determined for which nutrients criteria should be set and what the level should be. More details are available elsewhere^(45)^.

### Graphical representation and general recommendations

The Wheel of Five, the Dutch national counselling model since 1953^(46)^, was retained for several reasons. First, about 75 % of dietitians in the Netherlands were using the Wheel of Five in 2011 (Netherlands Nutrition Centre, unpublished results). Second, consumer research indicated that in 2015 the Wheel of Five was known by 92 % of the Dutch people, whereas 61 % knew its recommendations (Netherlands Nutrition Centre, unpublished results). The Wheel of Five was adapted to the newly derived recommended daily amounts of food groups and its design updated. Three different concepts, as well as several variants of the finally chosen concept, were tested by consumers and dietitians and discussed with experts on nutrition behaviour and communication. Additionally, general dietary recommendations were formulated and visualised.

## Results

### Results of optimisation calculations

Table 4 shows the results of the optimisation calculations in grams for various food groups. For children aged 1–8 years and girls aged 14–18 years, the optimisation calculations did not provide a result. For these groups, it was not possible to meet the constraints for essential nutrients within the constraints for energy. For the other age and gender groups, the model provided dietary patterns that met all constraints. There were different solutions for the different age and gender groups. For some food groups, there was no or little variation in optimised amounts across age and gender groups, e.g. for fruit (all 200 g) and fish and shellfish (range 15–18 g). Other food groups showed a large variation in optimised amounts, e.g. for vegetables (range 200–700 g) and drinks (range 487–3524 g).

### Recommended daily amounts for food groups

The results of the optimised dietary patterns served as a basis for deriving the recommended daily amounts for food groups (Tables 5 and 6). To send consistent and understandable messages to consumers, the results of the optimisation calculations were converted from grams to practical quantities or serving sizes. The outcome of the optimisation calculations, the HCNL advice on food groups and nutrients, closest adherence to current consumption, environmental impact and serving size were all taken into account. For example, for vegetables the amount in the optimised diets varied between 200 and 700 g for different target groups. The HCNL recommendation of at least 200 g of vegetables daily resulted in a somewhat lower vitamin A and folic acid provision in specific subgroups (results not shown). For most population groups, the optimisation calculations resulted in amounts between 235 and 271 g (Table 4). As we use 50 g of vegetables as the visualisation of a serving for consumers, we decided to recommend 250 g vegetables/d.

Table 7 shows the provision of nutrients by the recommended daily amounts of foods. These amounts provided about 85 % of the energy needed and, except for a few cases, at least 100 % of the nutrient DRV. The implication of each exception was evaluated, taking account of the difference with the DRV and the current intake of the specific nutrient. More detailed information is available^(38)^. If applicable, specific points of attention were defined for recommendations to consumers in case of an intake below the DRV, e.g. the use of sufficient leafy green vegetables to provide (pro)vitamin A, the consumption of wholegrain cereals to provide dietary fibre and the consumption of sufficient milk products to provide calcium. For young children and women of childbearing age having an iron intake below the DRV (Table 7), the advice is to consume foods that are naturally rich in iron and to use combinations of foods to enhance iron absorption (fruits and cereal products)^(47)^. Given those specific recommendations, experts expressed no concerns about nutrient adequacy for people who consume the recommended daily amounts of foods.

**Table 7:** Daily amounts of nutrients delivered by the daily recommended amounts for foods in the Wheel of Five per age and gender*,†

Nutrient (unit per day)	1–3 years		4–8 years		9–13 years		14–18 years		19–30 years		31–50 years		51–69 years		≥ 70 years		Pregnant	Lactating
	M	F	M	F	M	F	M	F	M	F	M	F	M	F	M	F		
Energy (kJ)	3787	3787	4682	4682	7544	6916	9364	7393	8740	7303	8740	7303	8527	6602	7745	6456	7824	9000
Energy (kcal)	905	905	1119	1119	1803	1653	2238	1767	2089	1745	2089	1745	2038	1578	1851	1543	1870	2151
Protein (g)	39	39	50	50	81	76	102	84	94	84	94	84	93	81	93	84	90	100
Total fatty acids (g)	34	34	41	41	65	61	78	65	81	64	81	64	81	58	67	55	68	86
SFA (g)	9	9	12	12	17	16	22	18	20	17	20	17	20	17	19	17	18	21
PUFA (g)	11	11	13	13	21	20	25	20	26	19	26	19	26	17	21	16	21	27
Linoleic acid (g)	9	9	11	11	18	17	21	17	22	16	22	16	22	14	17	13	18	23
*Tran*s-fatty acids (g)	0·3	0·3	0·3	0·3	0·5	0·4	0·6	0·5	0·6	0·5	0·6	0·5	0·6	0·5	0·6	0·5	0·5	0·5
α-Linolenic acid (g)	1·1	1·1	**1·5**	1·5	2·6	2·4	3·1	2·4	3·3	2·4	3·3	2·4	3·2	2·2	3·3	2·5	2·6	3·2
EPA + DHA (mg)	193	193	222	222	397	395	406	397	399	389	399	389	399	386	405	395	393	406
Cholesterol (mg)	83	83	122	122	158	157	178	168	163	162	163	162	166	168	173	171	167	172
Total carbohydrate (g)	104	104	127	127	208	186	262	197	229	193	229	193	217	168	201	163	207	226
Mono- and disaccharides (g)	37	37	39	39	56	55	66	57	54	52	54	52	57	57	61	59	–	–
Dietary fibre (g)	13	13	18	18	29	26	**36**	28	**35**	**30**	**35**	30	**33**	25	31	25	32	35
Water (g)	1244	1244	1573	1573	2202	1967	2701	2168	2636	2194	2636	2194	2578	2127	2586	2121	2615	2638
Vitamin A (µg)	416	416	491	491	661	633	**865**	758	**849**	707	**849**	707	**857**	724	913	783	**738**	**769**
Vitamin D (µg)	3	3	3	3	4	4	5	4	5	4	5	4	5	4	5	4	4	5
Vitamin E (mg)	8	8	10	10	15	14	18	14	19	14	19	14	18	13	15	12	15	18
Vitamin K (µg)	109	109	140	140	186	181	255	239	273	250	273	250	273	251	226	218	255	260
Thiamin (mg)	0·7	0·7	0·8	0·8	1·2	1·2	1·5	1·2	1·4	1·2	1·4	1·2	1·3	1·1	1·2	**1·0**	**1·3**	**1·5**
Riboflavin (mg)	1·0	1·0	1·1	1·1	1·6	1·6	2·1	1·7	1·7	1·5	1·7	1·5	1·8	1·7	1·9	1·8	1·6	1·7
Niacin (mg)	9	9	10	10	17	16	21	17	21	19	21	19	20	17	19	16	20	23
Vitamin B_6_ (mg)	1·0	1·0	1·2	1·2	1·9	1·8	2·3	1·9	2·1	1·8	2·1	1·8	2·0	1·7	2·0	1·7	2·0	2·1
Folate (µg)	135	135	189	189	293	273	375	311	376	328	376	328	370	310	388	337	**347**	**377**
Vitamin B_12_ (mg)	2·3	2·3	2·7	2·7	4·1	4·0	5·1	4·3	4·0	3·9	4·0	3·9	4·3	4·4	4·8	4·7	4·0	4·1
Vitamin C (mg)	38	38	50	50	74	73	91	85	97	97	97	97	96	95	103	100	97	98
Calcium (mg)	592	592	792	792	**1101**	**1063**	1532	1258	1253	1178	1253	1178	1331	1325	1470	1415	1216	1257
Phosphorus (mg)	806	806	1030	1030	1600	1515	2053	1677	1839	1682	1839	1682	1856	1664	1922	1756	1764	1974
Magnesium (mg)	203	203	249	249	402	371	498	400	488	428	488	428	473	376	446	380	460	543
Sodium (mg)	721	721	962	962	1540	1356	2015	1494	1914	1493	1914	1493	1846	1350	1645	1362	1671	1849
Potassium (mg)	**1715**	**1715**	2082	2082	3257	3099	4056	3392	3860	3568	3860	3568	3835	3465	3912	3512	3781	4049
Iron (mg)	**5**	**5**	**6**	**6**	**10**	**9**	13	**10**	13	**11**	13	**11**	12	9	11	9	**12**	**14**
Zinc (mg)	**5**	**5**	7	7	11	10	14	12	13	12	13	12	13	11	13	12	12	14
Selenium (µg)	26	26	33	33	53	50	64	55	61	56	61	56	59	50	**53**	48	59	70
Copper (mg)	0·7	0·7	0·9	0·9	1·4	1·3	1·7	1·4	1·7	1·5	1·7	1·5	1·7	1·3	1·4	1·2	1·6	2·0
Iodine (µg)	102	102	123	123	205	180	258	185	243	182	243	182	236	167	213	174	208	233

* Daily recommended amounts of foods deliver about 85 % of the energy requirements.

† Figures in bold do not reach the Dietary Reference Values of the Health Council of the Netherlands^(21)^.

#### Recommendations for target groups with higher energy requirements and different food preferences

##### Higher energy needs

Recommendations for pregnant and breast-feeding women were based on the recommendations for women aged 19–50 years (Table 6), fitting into the habitual dietary pattern^(22)^. To meet the higher nutrient and energy requirements for pregnant and breast-feeding women^(21,31)^ the recommendation is to consume up to two extra slices of bread with margarine and 25 g extra meat/d, depending on energy needs and activity level. For breast-feeding women, an extra 25 g nuts/d is recommended to meet their higher nutrient and energy requirements^(21,31)^. Additionally, the advice for pregnant women is to use folic acid supplements in the most vulnerable period of pregnancy^(48)^ and vitamin D, in line with HCNL^(41)^.

The physical activity level value used for active groups resulted in a 9–15 % higher energy requirement. For these groups, we drafted the advice to consume more plant-based foods like bread, wholegrain cereals, legumes and nuts to meet this extra energy requirement.

##### No meat

For those with a dietary pattern without meat, but including fish, a dietary pattern similar to that in Tables 5 and 6 is recommended in which the meat is replaced by a combination of nuts (2 × 25 g/week extra), legumes (135 g/week extra) and eggs (1 egg/week extra). Nutrient provision of these recommendations was evaluated (data not shown; see elsewhere^(38)^ for more details), which resulted in specific recommendations to consume foods naturally rich in iron, to use sufficient dairy and wholegrain products and to consume meat replacements with sufficient protein and enriched with iron and thiamin or vitamin B_12_.

##### Non-Western dietary patterns

When applying the recommended daily amounts for food groups as shown in Table 6 for people with Turkish, Moroccan and Surinamese backgrounds using their own typical products, the DRV for α-linoleic acid, vitamin A and vitamin D were not met (results available elsewhere^(38)^). In order to provide sufficient nutrients, specific recommendations for foods were drafted for these subgroups, like to use of leafy green vegetables and margarines for (pro)vitamin A and to choose fats and oils rich in α-linoleic acid. Additionally, for these subgroups HCNL advises the daily use of vitamin D supplements^(41)^.

### Criteria and recommendations for foods outside the Wheel of Five

Our calculations showed that energy, SFA and salt were limiting factors for foods outside the Wheel of Five. That is, for these factors, the intake through the recommended daily amounts of food groups was already relatively close to the maximum intake levels; for instance, about 20 % for SFA (women aged 31–50 years) and 20 % for salt (men aged 31–50 years)^(38)^. Criteria for these factors to discriminate between foods that could be consumed at least three times daily and foods that should be consumed exceptionally were: 314 kJ (75 kcal), 1·7 g SFA and 0·5 g salt per serving. With these criteria, maximum recommendations for energy, SFA and salt were not exceeded by three daily choices, and left some room for weekly choices (data not shown; see elsewhere^(45)^ for more details). Sugar-containing beverages like soft drinks and juices have their own, very distinctive criterion (16·7 kJ (4 kcal) per 100 ml) to distinguish between daily and weekly choices. Recommendations for foods outside the Wheel of Five are: ‘Consume daily choices no more than three to five times per day, and weekly choices no more than three times a week’.

### Graphical representation and general recommendations

**Fig. 2:**
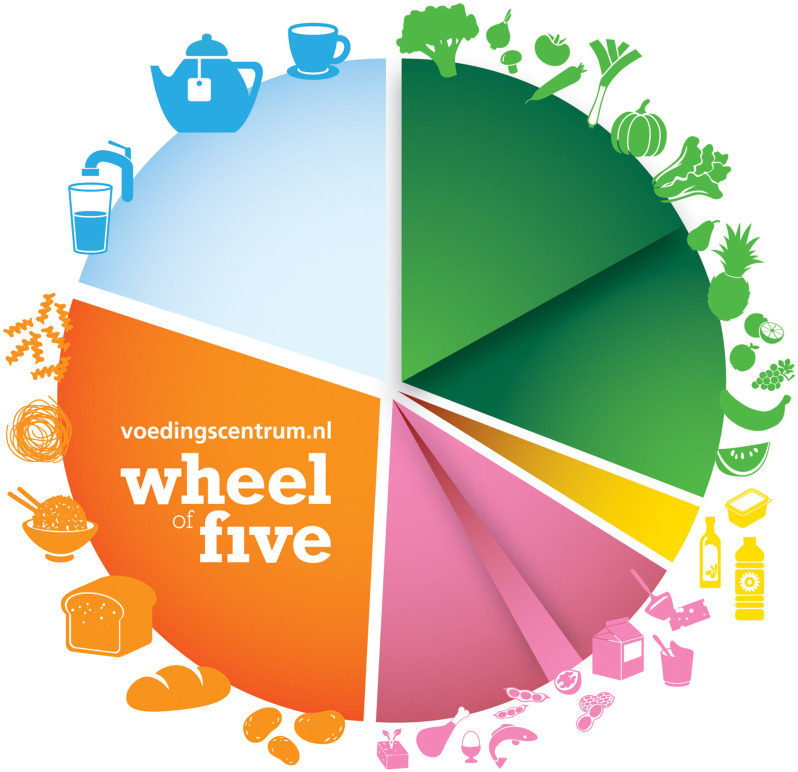
Wheel of Five: graphical representation of the food-based dietary guidelines for the Netherlands

The Dutch national dietary-counselling model is the Wheel of Five (Fig. 2). It includes five sections representing the combinations of food groups given in Tables 5 and 6. The graphical size of each section was determined as the ratio of the recommended amounts (in grams) for adult women, except for drinks (this was pre-set as one-fifth of the Wheel). The icons represent the food groups in each section. To represent each food group, the more environmentally friendly options were chosen. For fruit and vegetables, for instance, icons were chosen for foods that are available year-round and have a low environmental impact according to the Fruit and Vegetable Calendar^(49)^.

The recommended dietary patterns for the different age and gender groups (Tables 5 and 6) were summarised in seven general recommendations for the Wheel of Five:

- Eat lots of fruit and vegetables.
- Consume mainly wholegrain products such as wholegrain bread, wholegrain pasta and brown rice.
- Eat less meat and more plant-based foods, and vary with fish, pulses, nuts, eggs and vegetarian products.
- Consume sufficient dairy products such as milk, yoghurt and cheese.
- Eat a handful of unsalted nuts daily.
- Consume soft and liquid spreadable fats and cooking fats.
- Drink sufficient amounts of tap water, tea and coffee.

**Fig. 3:**
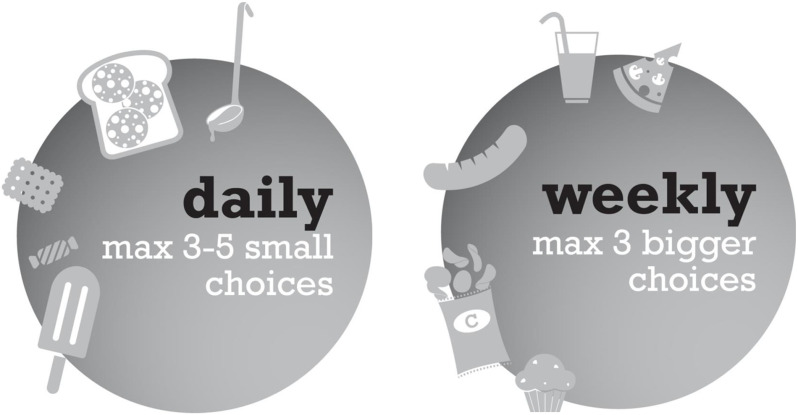
Graphical representation of recommendations for products outside the Wheel of Five for daily choices (left) and weekly choices (right)

Figure 3 shows the graphical representation for foods outside the Wheel of Five. In all cases, the general advice is that these foods should neither be eaten too often nor in large quantities. It is recommended to use small servings, defined by the energy, SFA and salt content per serving, to prevent undesirable weight gain.

## Discussion

The present paper describes the process, choices made and results of the development of the FBDG for the Netherlands. A consumption pattern in line with these guidelines reduces the risk of major chronic diseases, supplies adequate amounts of energy and nutrients, and can reduce the environmental impact compared with the mean current consumption. The process described in the present paper combined model and data-based elements with expert knowledge and common sense. The FBDG for the Netherlands consist of a graphical representation in the Wheel of Five, a set of seven general dietary guidelines, and quantitative recommended dietary patterns for several target groups. In terms of the general guidelines, there is a large similarity with the FBDG for other countries. Most FBDG promote an abundant consumption of fruit, vegetables and wholegrain cereals, and a limited consumption of products rich in SFA, TFA, simple sugars and salt^(3,50)^. A plate or circle as a graphical representation is also used in many other countries, as is the pyramid shape. The type of shape for practical nutrition communication does not play a major role in the effectiveness or efficiency of this communication^(51)^.

A consistent set of quantitative guidelines for a broad range of target groups is typical for the FBDG for the Netherlands. Apart from the usual subgroups by age and gender, and pregnant and breast-feeding women, these cover persons with culturally different diets because of immigrant backgrounds or specific dietary regimens (pescatarians). Few other FBDG are so broadly developed. In Europe, Italy and Albania include advice for menopausal women, and the UK for Asians^(3)^. In the Americas, some countries include specific dietary guidelines for vegetarians (Canada, Brazil, Puerto Rico and the USA), indigenous people (Canada and Venezuela), inhabitants of rural areas (Colombia and Peru), victims of violence (Colombia) and pregnant teenagers (Peru and Cuba)^(50)^. Target-group-specific guidelines are important because of differences in dietary requirements and in order to make the messages more culturally acceptable. The latter was also the reason why the current diet was chosen as an optimisation criterion in the optimisation modelling step of the Wheel of Five process.

A shift from the current Dutch food consumption pattern to a food pattern according to the Wheel of Five will result in a higher consumption of vegetables, fruits, wholegrain foods, nuts, fish and legumes, whereas the consumption of foods with a negative impact on health would be lower^(52)^. These changes in dietary pattern are associated with a reduction of the risk of chronic diseases and thus will result in health gain^(20)^. Kromhout *et al*.^(20)^ argue that the maximum health gain cannot be quantified. They showed that relative risks per food group are of the order of 10–20 %, but indicate that because of the correlations among food groups, the effects are not additive. However, the results of the PREDIMED (Prevención con Dieta Mediterránea; Prevention with a Mediterranean Diet) study suggest that the more the guidelines are adhered to, the greater the health gain compared with findings of cohort studies on dietary patterns^(53)^.

Despite the substantial evidence base showing the need and possibilities for aligning health and environmental objectives, only a few countries have so far included environmental sustainability in their FBDG. An inventory of the FAO published in 2016 identified Germany, Brazil, Sweden and Qatar^(7)^. More recently, the guidelines of Belgium for the Flemish population and for the UK also included environmental sustainability^(8,54)^. There are a number of factors that affect the sustainability of our food system, for example GHGE, land use and water use, but also pesticide use, animal welfare and food waste^(55–59)^. Van Dooren *et al*. evaluated fifty-five documented assessment methods of indicators for environmental impact and showed that the majority of the indicators cannot be used to assess the environmental impacts of diets because there is a lack of reliable data or internationally adapted methods and support. They concluded that GHGE and land use cover most of the environmental impact of diets^(58)^. GHGE is far and away the most commonly used indicator^(60)^. GHGE is also strongly correlated with land use, water use, acidification, freshwater eutrophication and marine eutrophication^(61)^. Per kilogram of product, meat and cheese have considerably higher GHGE compared with plant-based foods, i.e. 12–13 *v*. <3 kg CO_2_-equivalents^(62)^. For this reason, we set a maximum level for animal food groups in our optimisation model as a proxy for GHGE. This is also in line with the HCNL recommendation to follow a diet with less animal-based foods^(20,26)^. For most age groups, our optimisation results were at the pre-set maximum for total meat and eggs. This shows that without applying these constraints, the outcome of the optimisation model most likely would have resulted in a higher recommended intake of these animal-based foods, thus in diets with higher GHGE, since these foods are rich sources of several essential nutrients.

We did not intend to create FBDG for diets with the least environmental impact possible. This would require optimisation modelling with minimising an indicator like GHGE as objective function. Studies that did optimise for minimal GHGE showed that high reductions in GHGE resulted in dietary patterns that were not acceptable and/or not nutritionally adequate^(63,64)^. These studies also showed that sustainable dietary patterns that meet dietary requirements for health could be reached without eliminating meat or dairy products. A food pattern according to the Wheel of Five recommends a maximum consumption of 500 g of meat weekly. Particularly for adult men, this means a significant reduction compared with their current observed average meat consumption of 930 g/week^(22)^. For adult women, who currently consume 615 g/week^(22)^, this reduction is less substantial. Recently, Van de Kamp *et al*.^(61)^ showed that the shift from the current Dutch diet to the recommended dietary pattern in the Wheel of Five reduces GHGE up to 13 % for men aged 31–50 years, whereas they increase slightly by 2–5 % for women. More substantial reductions in GHGE are achieved with a further reduction of meat and replacing it by nuts, legumes and eggs. Alternatively, substantial reductions can be achieved by consuming only foods and beverages with relatively low GHGE within each food group, such as drinking tap water, coffee and tea and limiting the consumption of highly processed foods^(61)^. In line with this, the Netherlands Nutrition Centre provides additional practical advice for consumers to help them to make more sustainable choices: for instance, by examples of weekly menus that include four daily menus with meat and three daily menus with meat alternatives; by the recommendation to eat local fruits and vegetables of the season^(65)^; as well as by practical recommendations to prevent food waste and providing information on animal welfare and sustainability labels.

The recommended amounts for foods in the Wheel of Five provide approximately 85 % of the average energy requirement. Consequently, consumers who adhere to these dietary patterns can supplement their diet with other foods that are not part of the Wheel of Five, for example with processed foods that do not fulfil the salt, sugar, fibre or fatty acid criteria of the Wheel of Five, and sweet and salty snacks. Obtaining on average 15 % of the energy requirement from foods outside the Wheel of Five is a much lower figure than the observed consumption level in the Netherlands of about two-thirds of energy intake^(45)^. Other FBDG also indicate that the consumption of products rich in salt, sugar and SFA should be limited^(8,14)^. To the best of our knowledge, the Wheel of Five guidelines are unique in the sense that they provide practical recommendations for the consumption frequency and serving sizes of foods with a simple distinction between foods that may be consumed on a daily basis or a weekly basis.

The development process and methods have several strengths and limitations. A transparent and structured procedure was followed that consisted of a combination of data and model-driven steps, complemented by independent expert-based decisions. The optimisation model ensured that the FBDG included adaptations to the current food consumption pattern that were as small as possible, meet the recommendations for food groups and nutrients, and limit consumption of animal products with a high environmental impact. Optimisation modelling is considered the preferred approach, since it captures the complexity of the diet as a whole^(66)^, and is applied by several other countries^(13–15)^. The use of an optimisation model requires a range of decisions that potentially can influence the outcome^(67)^. Examples are the definition of food groups and their nutrient composition, the criteria for Wheel of Five food groups, the type of optimisation function (quadratic, linear) and constraints for food groups. As internal validity check, we performed a sensitivity analyses for one of the age and sex groups to study the impact of these choices in the optimisation modelling. Results of nine different scenarios were positively correlated with a reference scenario (Spearman’s *r* ranged from 0·62 to 0·98 with an average of 0·87; data not shown). The lowest correlation was observed in a scenario where all food groups were given a nutrient composition that was healthier, such as higher in essential nutrients and lower in SFA, TFA, sodium or sugar, for example. Among others, this resulted in lower amounts of vegetables and fruits compared with the reference scenario (136 g/d *v*. 211 g/d for vegetables and 88 g/d *v*. 126 g/d for fruit). Given the different results with different choices, it is essential that the process and decisions are transparent and documented.

Another limitation was that the optimisation model did not give a solution for some of the target groups. For the Wheel of Five derivation, this was not a problem because we could extrapolate recommendations from adjacent age groups. If no solution had been found for many or all groups, however, an alternative optimisation model approach that searched for a diet that violated the constraints as little as possible would have been preferable^(68)^. A disadvantage of the optimisation model used was that it gave only one optimal solution without providing an insight into other, slightly less optimal solutions. As a consequence, some optimisation results were inconsistent across the target groups, for example daily amounts of wholegrain products for adult men varied from 82 g/d for the over 70s to 200 g/d for men aged 19–30 years. A similar observation was made in a Japanese study, which for example recommended daily amounts of 35 and 164 g of wholegrain products for men aged 30–49 and aged 50–69 years, respectively^(13)^. Overall, developing FBDG remains a combination of science-based and expert-based decisions. Therefore, transparency of the process is warranted.

As indicated before, the FBDG for the Netherlands show a large similarity with the FBDG for other countries. They consistently promote an abundant consumption of fruit, vegetables and whole-grain cereals, and a limited consumption of products rich in SFA, TFA, simple sugars and salt^(3,50)^, showing good external validity. This is also the case when comparing the FBDG for the Netherlands with FBDG for other countries that integrated health and sustainability. Like our recommendations, these FBDG recommend to consume more plant-based foods, fruits and vegetables, to limit the amount of red and processed meat, and to consume (low-fat, unsweetened) milk and dairy products^(7)^. Sweden and Qatar set maximum recommendations for red and processed meat at 500 g/week^(7)^. This is higher than our recommendations of 500 g total meat (of which maximum 300 g red meat) per week. Recently, Willett *et al*.^(69)^ presented a dietary pattern that integrated health and sustainability, aiming at feeding the global population in 2050 within the planetary boundaries. This dietary pattern was characterised by mean recommended intakes of food groups and ranges around the mean to meet e.g. regional or cultural differences. Although our recommendations for fish, legumes and nuts are lower than the mean value given in this dietary pattern, whereas our recommendations for total meat, dairy products and potatoes are higher, our food group recommendations are, except for potatoes, within the indicated ranges. As described before, we stimulate consumers towards a more plant-based, less animal-based food pattern.

Dietary guidelines are a key component of a coherent food policy and are the basis for the development of policies intended to shift consumption patterns in healthier and more environmentally sustainable directions. They need to be widely communicated to health professionals and the general public. They also need to be linked to other food policies and interventions^(7)^, such as food reformulation, measures to create healthier food environments, and regulations on food marketing and advertising. Dissemination of the Wheel of Five and its recommendations to the general public is enhanced by means of repeated, targeted communications via diverse media channels, social media, tools, apps, cookery books, brochures and campaigns. Important strategies include changing dietary patterns in small steps and improving food literacy. Consumers are assisted by several tools and apps to adapt the diet to their personal situation, preferences and needs (type of work, activities during leisure time, etc.). Little is known about the efficiency of FBDG on a public health level^(70)^. Although a consumer survey in 2017 indicated that 96 % of Dutch consumers were aware of the Wheel of Five and 64 % indicate to understand it (Netherlands Nutrition Centre, unpublished results), it is important to monitor the effects of such an integrated approach with food consumption survey data.

## Conclusion

In conclusion, based on an optimisation model, scientific evidence, information on dietary patterns and expert knowledge, we derived FBDG for a wide range of target groups. The Wheel of Five is a key food-counselling model that can help Dutch consumers to make their diets healthier and more environmentally sustainable.

## Supplementary material

For supplementary material accompanying this paper visit https://doi.org/10.1017/S1368980019001435. click here to view supplementary material
