# Supplementary material for: Development of healthy and sustainable food-based dietary guidelines for the Netherlands
Source: Public Health Nutr. 2019 Jul 2;22(13):2419–35. doi: 10.1017/S1368980019001435 (PMC7083597; doi:10.1017/S1368980019001435)
Supplement: Supplementary file 1 [file S1368980019001435sup001.docx]

| *Supplementary Table 1. List of minimum and maximum constraints (expressed as unit per day) for energy and nutrients used in the optimisation calculations for the development of FBDG for the Netherlands, specified per sex and age.^*^* ^†^ | | | | | | | | | | | | | | | | | |
| --- | --- | --- | --- | --- | --- | --- | --- | --- | --- | --- | --- | --- | --- | --- | --- | --- | --- |
|  |  |  |  |  |  |  |  |  |  |  |  |  |  |  |  |  |  |
|  | **Men** | **1-3 years** | | **4-8 years** | | **9-13 years** | | **14-18 years** | | **19-30 years** | | **31-50 years** | | **51-69 years** | | **> 70 years** | |
| Constraints | Unit/day | Min | Max | Min | Max | Min | Max | Min | Max | Min | Max | Min | Max | Min | Max | Min | Max |
|  |  |  |  |  |  |  |  |  |  |  |  |  |  |  |  |  |  |
| Energy^‡^ | kcal | 935 | 945 | 1410 | 1420 | 2140 | 2150 | 2750 | 2760 | 2780 | 2790 | 2650 | 2660 | 2420 | 2430 | 2190 | 2200 |
| Protein^§^ | g | 12 | 47 | 18 | 89 | 32 | 134 | 55 | 173 | 63 | 174 | 60 | 166 | 61 | 152 | 60 | 138 |
| Carbohydrates^§^ | g | 105 | 165 | 159 | 249 | 241 | 376 | 275 | 483 | 278 | 488 | 265 | 466 | 242 | 425 | 219 | 385 |
| Dietary fibre | g | 13 | `- | 20 | - | 30 | - | 39 | - | 40 | - | 38 | - | 34 | - | 31 | - |
| Total fat^§^ | g | 26 | 42 | 31 | 63 | 48 | 96 | 61 | 123 | 62 | 124 | 59 | 118 | 54 | 108 | 49 | 98 |
| Saturated fatty acids^§^ | g | 0 | 11 | 0 | 16 | 0 | 24 | 0 | 31 | 0 | 31 | 0 | 30 | 0 | 27 | 0 | 24 |
| Linoleic acid | g | 2,1 | - | 3,1 | - | 4,8 | - | 6,1 | - | 6,2 | - | 5,9 | - | 5,4 | - | 4,9 | - |
| Alpha linoleic acid | g | 1,0 | - | 1,6 | - | 2,4 | - | 3,1 | - | 3,1 | - | 2,9 | - | 2,7 | - | 2,4 | - |
| Docosahexaenoic acid & eicosapentaenoic acid | g | 150 | - | 150 | - | 150 | - | 200 | - | 200 | - | 200 | - | 200 | - | 200 | - |
| Trans fatty acids^§^ | g | 0,0 | 1,1 | 0 | 1,6 | 0 | 2,4 | 0 | 3,1 | 0 | 3,1 | 0 | 3,0 | 0 | 2,7 | 0 | 2,4 |
| Alcohol^‖^ | g | 0 | 0 | 0 | 0 | 0 | 0 | 0 | 0 | 0 | 0 | 0 | 0 | 0 | 0 | 0 | 0 |
| Retinol Activity Equivalents | ug | 350 | - | 400 | - | 600 | - | 900 | - | 900 | - | 900 | - | 900 | - | 900 | - |
| Retinol | ug | 0 | 800 | 0 | 1100 | 0 | 1500 | 0 | 2600 | 0 | 3000 | 0 | 3000 | 0 | 3000 | 0 | 3000 |
| Vitamin B1 | mg | 0,3 | - | 0,5 | - | 0,8 | - | 1,1 | - | 1,1 | - | 1,1 | - | 1,1 | - | 1,1 | - |
| Vitamin B2 | mg | 0,5 | - | 0,7 | - | 1 | - | 1,5 | - | 1,5 | - | 1,5 | - | 1,5 | - | 1,5 | - |
| Nicotinic acid | mg | 4 | - | 7 | - | 11 | - | 17 | - | 17 | - | 17 | - | 17 | - | 17 | - |
| Vitamin B6 | mg | 0,4 | 5,0 | 0,7 | 7,0 | 1,1 | 10 | 1,5 | 20 | 1,5 | 25 | 1,5 | 25 | 1,8 | 25 | 1,8 | 25 |
| Folate Equivalents | ug | 85 | - | 150 | - | 225 | - | 300 | - | 300 | - | 300 | - | 300 | - | 300 | - |
| Folate | ug | 0 | 200 | 0 | 300 | 0 | 400 | 0 | 800 | 0 | 1000 | 0 | 1000 | 0 | 1000 | 0 | 1000 |
| Vitamin B12 | ug | 0,7 | - | 1,3 | - | 2 | - | 2,8 | - | 2,8 | - | 2,8 | - | 2,8 | - | 2,8 | - |
| Vitamin C | mg | 30 | - | 40 | - | 50 | - | 75 | - | 75 | - | 75 | - | 75 | - | 75 | - |
| Vitamin D | ug | 3,5 | 50,0 | 3,5 | 50,0 | 3,5 | 50 | 3,5 | 100 | 3,5 | 100 | 3,5 | 100 | 3,5 | 100 | 3,5 | 100 |
| Vitamin E | mg | 5 | 100 | 6 | 120 | 8 | 160 | 10 | 260 | 10 | 300 | 10 | 300 | 10 | 300 | 10 | 300 |
| Vitamin K | ug | 30 | - | 55 | - | 60 | - | 75 | - | 120 | - | 120 | - | 120 | - | 120 | - |
| Calcium | mg | 500 | 2500 | 700 | 2500 | 1200 | 2500 | 1200 | 2500 | 1000 | 2500 | 1000 | 2500 | 1100 | 2500 | 1200 | 2500 |
| Phosphorus | mg | 470 | - | 540 | - | 700 | - | 700 | - | 600 | - | 600 | - | 600 | - | 600 | - |
|  |  |  |  |  |  |  |  |  |  |  |  |  |  |  |  |  |  |
|  |  |  | |  | |  | |  | |  | |  | |  | |  | |
|  | **Men** | **1-3 years** | | **4-8 years** | | **9-13 years** | | **14-18 years** | | **19-30 years** | | **31-50 years** | | **51-69 years** | | **> 70 years** | |
| Constraints | Unit/day | Min | Max | Min | Max | Min | Max | Min | Max | Min | Max | Min | Max | Min | Max | Min | Max |
|  |  |  |  |  |  |  |  |  |  |  |  |  |  |  |  |  |  |
| Iron | mg | 8 | - | 9 | - | 11 | - | 11 | - | 9 | - | 9 | - | 9 | - | 9 | - |
| Sodium^‖^ | mg | 0 | 1200 | 0 | 1800 | 0 | 2400 | 0 | 2400 | 0 | 2400 | 0 | 2400 | 0 | 2400 | 0 | 2400 |
| Potassium | mg | 1800 | - | 2000 | - | 3300 | - | 3500 | - | 3500 | - | 3500 | - | 3500 | - | 3500 | - |
| Magnesium | mg | 120 | - | 200 | - | 280 | - | 350 | - | 350 | - | 350 | - | 350 | - | 350 | - |
| Zinc | mg | 6 | 7 | 7 | 10 | 11 | 13 | 12 | 22 | 9 | 25 | 9 | 25 | 9 | 25 | 9 | 25 |
| Selenium | ug | 25 | 60 | 30 | 90 | 40 | 130 | 60 | 250 | 60 | 300 | 60 | 300 | 60 | 300 | 60 | 300 |
| Copper | mg | 0,4 | 1,0 | 0,5 | 2,0 | 0,7 | 3 | 0,9 | 4 | 0,9 | 5 | 0,9 | 5 | 0,9 | 5 | 0,9 | 5 |
| Iodine | ug | 90 | 200 | 120 | 250 | 150 | 300 | 150 | 500 | 150 | 600 | 150 | 600 | 150 | 600 | 150 | 600 |
|  |  |  |  |  |  |  |  |  |  |  |  |  |  |  |  |  |  |
|  | **Women** | **1-3 years** | | **4-8 years** | | **9-13 years** | | **14-18 years** | | **19-30 years** | | **31-50 years** | | **51-69 years** | | **> 70 years** | |
| Constraints | Unit/day | Min | Max | Min | Max | Min | Max | Min | Max | Min | Max | Min | Max | Min | Max | Min | Max |
|  |  |  |  |  |  |  |  |  |  |  |  |  |  |  |  |  |  |
| Energy^‡^ | kcal | 850 | 860 | 1310 | 1320 | 1950 | 1960 | 2130 | 2140 | 2010 | 2020 | 1930 | 1940 | 1780 | 1790 | 1740 | 1750 |
| Protein^§^ | g | 11 | 43 | 16 | 83 | 29 | 123 | 43 | 134 | 45 | 126 | 43 | 121 | 45 | 112 | 48 | 109 |
| Carbohydrates^§^ | g | 96 | 151 | 147 | 231 | 219 | 343 | 213 | 375 | 201 | 354 | 193 | 340 | 178 | 313 | 174 | 306 |
| Dietary fibre | g | 12 | - | 19 | - | 28 | - | 30 | - | 29 | - | 27 | - | 25 | - | 25 | - |
| Total fat^§^ | g | 24 | 38 | 29 | 59 | 43 | 87 | 47 | 95 | 45 | 90 | 43 | 86 | 40 | 80 | 39 | 78 |
| Saturated fatty acids^§^ | g | 0 | 10 | 0 | 15 | 0 | 22 | 0 | 24 | 0 | 22 | 0 | 22 | 0 | 20 | 0 | 19 |
| Linoleic acid | g | 1,9 | - | 2,9 | - | 4,3 | - | 4,7 | - | 4,5 | - | 4,3 | - | 4,0 | - | 3,9 | - |
| Alpha linoleic acid | g | 0,9 | - | 1,5 | - | 2,2 | - | 2,4 | - | 2,2 | - | 2,1 | - | 2,0 | - | 1,9 | - |
| Docosahexaenoic acid & eicosapentaenoic acid | g | 150 | - | 150 | - | 150 | - | 200 | - | 200 | - | 200 | - | 200 | - | 200 | - |
| Trans fatty acids^§^ | g | 0,0 | 1,0 | 0 | 1,5 | 0 | 2,2 | 0 | 2,4 | 0 | 2,2 | 0 | 2,2 | 0 | 2,0 | 0 | 1,9 |
| Alcohol^‖^ | g | 0 | 0 | 0 | 0 | 0 | 0 | 0 | 0 | 0 | 0 | 0 | 0 | 0 | 0 | 0 | 0 |
| Retinol Activity Equivalents | ug | 350 | - | 400 | - | 600 | - | 700 | - | 700 | - | 700 | - | 700 | - | 700 | - |
| Retinol | ug | 0 | 800 | 0 | 1100 | 0 | 1500 | 0 | 2600 | 0 | 3000 | 0 | 3000 | 0 | 3000 | 0 | 1500 |
| Vitamin B1 | mg | 0,3 | - | 0,5 | - | 0,8 | - | 1,1 | - | 1,1 | - | 1,1 | - | 1,1 | - | 1,1 | - |
| Vitamin B2 | mg | 0,5 | - | 0,7 | - | 1 | - | 1,1 | - | 1,1 | - | 1,1 | - | 1,1 | - | 1,1 | - |
| Nicotinic acid | mg | 4 | - | 7 | - | 11 | - | 13 | - | 13 | - | 13 | - | 13 | - | 13 | - |
|  |  |  |  |  |  |  |  |  |  |  |  |  |  |  |  |  |  |
|  | **Women** | **1-3 years** | | **4-8 years** | | **9-13 years** | | **14-18 years** | | **19-30 years** | | **31-50 years** | | **51-69 years** | | **> 70 years** | |
| Constraints | Unit/day | Min | Max | Min | Max | Min | Max | Min | Max | Min | Max | Min | Max | Min | Max | Min | Max |
|  |  |  |  |  |  |  |  |  |  |  |  |  |  |  |  |  |  |
| Vitamin B6 | mg | 0,4 | 5,0 | 0,7 | 7 | 1,1 | 10 | 1,5 | 20 | 1,5 | 25 | 1,5 | 25 | 1,5 | 25 | 1,5 | 25 |
| Folate Equivalents | ug | 85 | - | 150 | - | 225 | - | 300 | - | 300 | - | 300 | - | 300 | - | 300 | - |
| Folate | ug | 0 | 200 | 0 | 300 | 0 | 400 | 0 | 800 | 0 | 1000 | 0 | 1000 | 0 | 1000 | 0 | 1000 |
| Vitamin B12 | ug | 0,7 | - | 1,3 | - | 2 | - | 2,8 | - | 2,8 | - | 2,8 | - | 2,8 | - | 2,8 | - |
| Vitamin C | mg | 30 | - | 40 | - | 50 | - | 75 | - | 75 | - | 75 | - | 75 | - | 75 | - |
| Vitamin D | ug | 3,5 | 50,0 | 3,5 | 50 | 3,5 | 50 | 3,5 | 100 | 3,5 | 100 | 3,5 | 100 | 3,5 | 100 | 3,5 | 100 |
| Vitamin E | mg | 5 | 100 | 6 | 120 | 7 | 160 | 8 | 260 | 8 | 300 | 8 | 300 | 8 | 300 | 8 | 300 |
| Vitamin K | ug | 30 | - | 55 | - | 60 | - | 75 | - | 90 | - | 90 | - | 90 | - | 90 | - |
| Calcium | mg | 500 | 2500 | 700 | 2500 | 1100 | 2500 | 1100 | 2500 | 1000 | 2500 | 1000 | 2500 | 1100 | 2500 | 1200 | 2500 |
| Phosphorus | mg | 470 | - | 540 | - | 700 | - | 700 | - | 600 | - | 600 | - | 600 | - | 600 | - |
| Iron | mg | 8 | - | 9 | - | 11 | - | 15 | - | 15 | - | 15 | - | 9 | - | 9 | - |
| Sodium^‖^ | mg | 0 | 1200 | 0 | 1800 | 0 | 2400 | 0 | 2400 | 0 | 2400 | 0 | 2400 | 0 | 2400 | 0 | 2400 |
| Potassium | mg | 1800 | - | 2000 | - | 2900 | - | 3100 | - | 3100 | - | 3100 | - | 3100 | - | 3100 | - |
| Magnesium | mg | 120 | - | 200 | - | 280 | - | 280 | - | 280 | - | 280 | - | 280 | - | 280 | - |
| Zinc | mg | 6 | 7 | 7 | 10 | 8 | 13 | 9 | 22 | 7 | 25 | 7 | 25 | 7 | 25 | 7 | 25 |
| Selenium | ug | 25 | 60 | 30 | 90 | 40 | 130 | 50 | 250 | 50 | 300 | 50 | 300 | 50 | 300 | 50 | 300 |
| Copper | mg | 0,4 | 1,0 | 0,5 | 2 | 0,7 | 3 | 0,9 | 4 | 0,9 | 5 | 0,9 | 5 | 0,9 | 5 | 0,9 | 5 |
| Iodine | ug | 90 | 200 | 120 | 250 | 150 | 300 | 150 | 500 | 150 | 600 | 150 | 600 | 150 | 600 | 150 | 600 |

* Minimum values are the dietary reference values (DRV) of the Health Council of the Netherlands (HCNL) ^(21)^, unless stated otherwise.

† Maximum values for micronutrients are EFSA’s safe upper levels^(27)^, unless stated otherwise.

‡ Minimum value for energy is 85% of HCNL recommended intake for energy (= EAR) for inactive persons^(31)^. PAL value for inactive persons is 1.4 for women and 1.5 for men ^(32)^. Maximum value for energy is + 10 kcal for optimisation purposes.

§ HCNL DRV ^(21)(31)^. For total fat, protein and carbohydrates recommendations are given in ranges (and energy %). The minimum value is the lower value of the recommended range; the maximum value the upper value of the recommended range. For trans fatty acids and saturated fatty acids HCNL recommends maximum amount. These are set as maximum value for these nutrients.

‖ Maximum values for alcohol and sodium are based on HCNL Dutch dietary guidelines ^(20)^.
